# SOCIAL SUPPORT NETWORKS AND LONELINESS: DIFFERENCES ACROSS RACIAL/ETHNIC GROUPS

**DOI:** 10.1093/geroni/igae098.3893

**Published:** 2024-12-31

**Authors:** Orah Burack, Wingyun Mak

**Affiliations:** The New Jewish Home, New York, New York, United States; The New Jewish Home, New York, New York, United States

## Abstract

Higher levels of social support (SS) has been associated with a number of positive health and well-being outcomes including lower levels of loneliness. Additionally, SS networks have been found to vary across race/ethnicity. Researchers have therefore begun to examine how specific aspects of SS differentially impact outcomes among racial/ethnic groups. In this study we examined whether visits with different types of SS providers (children, siblings, friends), impact loneliness differentially across race/ethnicity. Data were drawn from the AARP Loneliness and Social Connections Survey with a national sample of community dwelling adults age 45+ years (M=61.84, SD=10.12; N=4040). Participants self-identified as White non-Hispanic (N=3149), Black non-Hispanic (N=420), or Hispanic (N=472). A series of hierarchical linear regressions (one for each racial/ethnic group) examined the relationship between loneliness and frequency of in-person visits from: children, siblings, or friends, after controlling for age, gender, marital status, and perceived health. While older, married, healthier adults reported less loneliness across all groups, differences were found by SS provider type. Less loneliness was significantly related to more visits from: siblings (β=.05, p<.001) and friends (β=.11, p<.001) for Whites (ΔR2=.03, p<.001); friends (β=.09, p=.008) for Hispanics (ΔR2=.02., p=.01); while for Black subjects there were similar size trends for children (β=.08, p=.08), siblings (β=.07, p=.03., p=.001), and friends (β=.07, p=.09). These results highlight that SS providers are not interchangeable, with differing impacts on loneliness across ethnicity/race. Implications and reasons for these findings will be discussed.

